# MRI quantified enlarged perivascular space volumes as imaging biomarkers correlating with severity of anxiety depression in young adults with long-time mobile phone use

**DOI:** 10.3389/fpsyt.2025.1532256

**Published:** 2025-02-20

**Authors:** Li Li, Yalan Wu, Jiaojiao Wu, Bin Li, Rui Hua, Feng Shi, Lizhou Chen, Yeke Wu

**Affiliations:** ^1^ Department of Radiology, Hospital of Chengdu University of Traditional Chinese Medicine, Chengdu, Sichuan, China; ^2^ Department of Research and Development, Shanghai United Imaging Intelligence Co., Ltd., Shanghai, China; ^3^ Department of Geriatrics, Hospital of Chengdu University of Traditional Chinese Medicine, Chengdu, China; ^4^ Department of Radiology, West China Hospital of Sichuan University, Chengdu, Sichuan, China; ^5^ Department of Stomatology, Hospital of Chengdu University of Traditional Chinese Medicine, Chengdu, China

**Keywords:** MRI, imaging biomarker, anxiety, depression, LTMPU, EPVS

## Abstract

**Introduction:**

Long-time mobile phone use (LTMPU) has been linked to emotional issues such as anxiety and depression while the enlarged perivascular spaces (EPVS), as marker of neuroinflammation, is closely related with mental disorders. In the current study, we aim to develop a predictive model utilizing MRI-quantified EPVS metrics and machine learning algorithms to assess the severity of anxiety and depression symptoms in patients with LTMPU.

**Methods:**

Eighty-two participants with LTMPU were included, with 37 suffering from anxiety and 44 suffering from depression. Deep learning algorithms were used to segment EPVS lesions and extract quantitative metrics. Comparison and correlation analyses were performed to investigate the relationship between EPVS and self-reported mood states. Training and testing datasets were randomly assigned in the ratio of 8:2 to perform radiomics analysis, where EPVS metrics combined with sex and age were used to select the most valuable features to construct machine learning models for predicting the severity of anxiety and depression.

**Results:**

Several EPVS features were significantly different between the two comparisons. For classifying anxiety status, eight features were selected to construct a logistic regression model, with an AUC of 0.819 (95%CI 0.573-1.000) in the testing dataset. For classifying depression status, eight features were selected to construct a K nearest neighbors model with an AUC value of 0.931 (95%CI 0.814-1.000) in the testing dataset.

**Discussion:**

The utilization of MRI-quantified EPVS metrics combined with machine-learning algorithms presents a promising method for evaluating severity of anxiety and depression symptoms in patients with LTMPU, which might introduce a non-invasive, objective, and quantitative approach to enhance diagnostic efficiency and guide personalized treatment strategies.

## 1 Introduction

The pervasive use of mobile phones in the modern era has transformed the way we live and interact. Their convenience and efficiency have made them an essential tool for young people. As of June 2022, nearly 1.05 billion people in China accessed the internet *via* mobile devices, accounting for 99.6% of internet users (1). Cell phone usage is highly prevalent among young adults (2), with many spending an average of 4.4 hours daily on their phones (3). While mobile phones offer undeniable benefits, their impact on mental health, particularly in relation to anxiety and depression, warrants attention. Long-time mobile phone use (LTMPU) is defined as using mobile phone ≥4 hours/day and previous studies have shown that LTMPU is associated with increased sleep disturbances and mental distress (4). In a recent meta-analysis, mobile phone addiction has been linked to higher levels of anxiety, depression, impulsivity, and poor sleep quality (5).

Anxiety disorders and depression are among the most prevalent mental health conditions, characterized by high rates of comorbidity and chronicity. Despite the significant burden on individuals and society, these conditions are often underdiagnosed and undertreated (6, 7). Differentiating between normal anxiety and clinical anxiety requires careful clinical judgment, considering severity, duration, persistence, and particularly the distress and impairment levels (6). Similarly, treatment effectiveness varies based on the severity of anxiety. A meta-analysis showed that individuals with mild or subthreshold depression had little or no benefit from therapeutic guidance while guided internet-based cognitive behavioral therapy (iCBT) demonstrated greater efficacy in moderate to severe cases (7). Therefore, an accurate assessment of anxiety and depression severity is crucial for effective treatment planning.

Currently, clinicians predominantly depend on subjective clinical observations, patient histories, and self-report questionnaires, such as the Hamilton Anxiety Scale and Hamilton Depression Scale, to evaluate symptoms and severity. However, these methods are not only time-consuming, but also subjective and difficult to replicate (8, 9). This has resulted in an escalating demand for objective and early detection methods to assess the severity of anxiety and depression.

Machine learning presents a promising approach for automating data analysis and establishing efficient and reproducible pipelines. Its application in psychiatry has grown rapidly in recent years, aiming to bridge the gap between group-level diagnostic or prognostic markers and clinical relevance (8). Specifically, the combination of machine learning and neuroimaging techniques, such as magnetic resonance imaging (MRI), has shown potential for detecting and predicting anxiety disorders and depression more accurately and efficiently (10, 11). Perivascular spaces (PVS) are fluid-filled areas surrounding blood vessels in the brain. They typically appear as linear, ovoid or rounded hyperintensities on T2 scans and are considered to be enlarged when their diameter exceeds 1 mm. Although enlarged perivascular spaces (EPVS) are typically considered a normal finding in subjects, recent research suggests that they may have significant clinical implications, particularly in relation to cerebrovascular and neurodegenerative diseases. Although research on the association between EPVSs and anxiety is limited, EPVSs are considered a marker of neuroinflammation, which is closely linked to mental illness (12). Studies have suggested that EPVSs in the centrum semiovale are associated with poststroke depression (13), and cerebral small-vessel disease features, including EPVSs and white matter hyperintensities, are risk factors for developing depression (12). Computational methods for quantifying EPVS parameters are now available (12).

This study aims to develop a predictive model utilizing MRI-quantified EPVS volumes and machine learning to assess anxiety and depression symptom severity in young adults with LTMPU. By exploring this novel approach, we hope to elucidate the potential correlation between EPVS and emotional disturbances in individuals addicted to mobile phone use.

## 2 Materials and methods

### 2.1 Participants

This study was a school-based cross-sectional study, which were conducted from October 2021 to May 2022. A total of 165 students and young teachers aged 18 to 50 years in a medical college in Wenjiang District, Chengdu, China were recruited in this study. Among them, 146 (88.5%) responded with valid data. Questionnaires were distributed to the students and young teachers during class period. This study was approved by the ethics committee of Hospital of Chengdu University of Traditional Chinese Medicine.

The inclusion criteria in this study were as follows: (a) with LTMPU. The duration of mobile phone use per day was obtained by the following question: How long do you usually spend on using mobile phone per day? The response categories for this question were: less than 2 hours, 2 to 4 hours, 4 to 6 hours, and more than 6 hours. LTMPU was defined as using mobile phone ≥4 hours per day in consideration of the recent findings (4); (b) ethnic Han; (c) free of any psychoactive medication at least 2 weeks before and during the study; (d) right-handedness assessed with the Edinburgh Handedness Inventory (14). Exclusion criteria in this study were as follows: (a) with coronavirus disease 2019 (COVID-19) infections; (b) with any significant neuropsychiatric disease or brain structural abnormality; (c) with MRI contraindications.

Furthermore, to evaluate mental status, all participants were asked to complete the Hamilton Anxiety (HAM-A) Scale and Hamilton Depression (HAM-D) Scale. The HAM-A was used to assess the severity of anxiety symptom. The total comprehensive score of HAM-A is in the range of 0 to 56. A HAM-A score ≤ 7 indicates no or minimal anxiety; 8–14 indicates mild anxiety; 15–23 indicates moderate anxiety; and ≥ 24 indicates severe anxiety (15, 16). The severity of depression symptom was assessed by HAM-D. A global HAM-D score of 0–54. A HAM-D score ≤ 7 indicates no depression; 8–16 indicates mild depression; 17–23 indicates moderate depression; and ≥ 24 indicates severe depression (17).

At baseline, 91 out of 146 participants (62.3%) reported using mobile phone ≥4 hours per day (LTMPU). Each participant with LTMPU completed informed written consent before undergoing magnetic resonance (MR) imaging (within two weeks after completing the scale). Nine participants were excluded because of MRI motion artifact. Finally, 82 participants with LTMPU were included. The power analysis was performed to justify the sample size (18). We hypothesized that Average_length_of_EPVS_in_left_frontal_lobe could be an effective factor in distinguishing between non-anxiety and anxiety groups. The statistical power for the given parameters was calculated using G*Power software (https://www.psychologie.hhu.de/arbeitsgruppen/allgemeine-psychologie-und-arbeitspsychologie/gpower). As shown in **Supplementary Figure S1A**, the sample sizes of group 1 (non-anxiety) and group 2 (anxiety) were 45 and 37, respectively. And the means of Average_length_of_EPVS_in_left_frontal_lobe for the two groups were 3.05 ( $\mu_{0}$ ) and 2.44 ( $\mu_{A}$ ), respectively, and the effect size ( $d=\frac{\mu_{A}-\mu_{0}}{\sigma }$ ) was 0.75. At a setting of α=0.05 , the statistical power ( 1−β ) could reach 0.906. Similarly, the Volume_of_EPVS_in_left_occipital_lobe classified non-depression and depression with statistical power of up to 0.947 for given parameters (α, sample sizes, means, and σ) (**Supplementary Figure S1B**).

### 2.2 MR imaging

All participants were examined using a 3.0 T whole body scanner (Discovery MR750, GE Healthcare, Milwaukee, WI) equipped with a 32-channel phased array head coil. T2-weighted images (T2WI) acquisition parameters were: TR = 5613 ms, TE =116 ms, slice thickness= 5.0 mm, slice spacing=1.5 mm, FOV = 26 mm. 3D T1-weighted imaging (T1WI) was acquired using spoiled gradient echo sequence with repetition time = 2.9 ms, echo time = 3.0 ms, inversion time = 450 ms, flip angle = 8°, slice thickness = 1 mm, matrix = 250 × 250, FOV = 22 cm × 22 cm.

### 2.3 Data preprocessing and EPVS quantification

**Figure 1:**
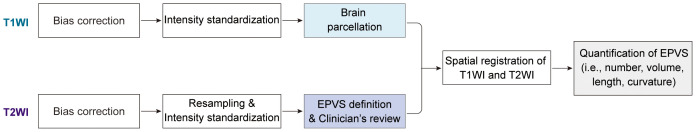
Schematics of the image processing and methods.

The image preprocessing procedure consisted of several steps, as illustrated in **Figure 1**. The details are outlined below (1): N4 bias field corrections were applied to both T1WI and T2WI to eliminate magnetic field inhomogeneity; (2) Grayscale values were standardized, with grayscale intensities normalized to the range of [-1, 1] by clipping the intensities at 0.1%-99.9%; (3) A deep learning model (VB-Net), embedded in an image analysis tool named uAI research portal (uRP, United Imaging Intelligence) (19), was employed to remove the skull from T1WI and segment the whole brain into 109 regions of interest (ROIs) based on the DK atlas (20). Subsequently, these regions were consolidated into 17 brain subregions as detailed in **Supplementary Table S1**, including bilateral frontal lobes, parietal lobes, occipital lobes, temporal lobes, basal ganglia, cerebellum, thalamus, centrum semiovale, and brainstem; (4) EPVS lesions were automatically segmented based on T2WI image with a corresponding mask generated through a built-in VB-Net model (21). The AI-generated masks were reviewed and amended by two experienced radiologists as needed. (5) T1WI and T2WI images were co-registered using a registration algorithm (22), and the segmentation mask of the T1WI space was transformed into T2WI space. (6) A series of quantitative metrics including number, volume, average length, and average curvature of EPVS lesions were computed for each brain subregion.

### 2.4 Radiomics analysis

To explore the ability of EPVS characteristics to predict anxiety and depression status, radiomics analysis including feature selection, model construction, and performance evaluation was performed *via* the uRP platform (23).

#### 2.4.1 Data grouping

Among 82 participants, 80% served as the training dataset, used for feature selection and model construction. The rest 20% served as the testing dataset, used to evaluate the robustness and generalizability of the model.

#### 2.4.2 Feature selection

A total of 70 EPVS quantitative features (i.e., total number, total volume, 4 features/subregion * 17 subregions), integrated with 2 clinical features (i.e., sex, age) served as the input to identify the most valuable biomarkers for clinical outcomes. Notably, feature standardization was first conducted to eliminate the effect of magnitudes between different features. Then, the least absolute shrinkage and selection operator (LASSO) regression was performed to select the most relevant feature combinations.

#### 2.4.3 Model construction

Based on the selected features, multiple machine learning algorithms (e.g., support vector machine [SVM], random forest [RF], logistic regression [LR], and K nearest neighbors [KNN]) were used to construct the classification models. For each classification task, we retained the model with the highest discriminative performance, where the LR model was used for the HAM-A classification and the KNN model for the HAM-D classification.

#### 2.4.4 Model evaluation

The performance of models was evaluated in the testing dataset, which could reflect the robustness and generalizability of models. The receiver operating characteristic (ROC) curve was first plotted, where the area under the curve (AUC) could be calculated quantitatively. Five metrics were calculated to evaluate the consistency between the actual label and predictive label, including accuracy, sensitivity, specificity, precision, and F1-score. These metrics were defined as follows (Equations 1-5):


(1) $$Accuracy=\;\frac{TP+TN}{TP+PF+TN+FN}$$

(2) $$Sensitivity=\;Recall=\;\frac{TP}{TP+FN\;}$$

(3) $$Specificity=\frac{TN}{TN+FP\;}$$

(4) $$Precision=\frac{TP}{TP+FP\;}$$

(5) $$F1score=\frac{2*Precision*Recall}{Precision+Recall}$$

where TP represented true positive, TN represented true negative, FP represented false positive, and FN represented false negative. Calibration curves were also used to compare the predictive output and the actual outcome. Finally, the decision curves were utilized to show the clinical net benefit for predicting outcomes.

### 2.5 Statistical analysis

The Shapiro-Wilk tests were used to check the normal distribution of continuous variables. For continuous variables that were approximately normally distributed, they were represented as mean ± standard deviation. For continuous variables with asymmetrical distributions, they were represented as median (25^th^, 75^th^ percentiles). Categorical variables were represented as counts (percentages), and compared using chi-square tests. To quantitatively compare the EPVS characteristics in the anxious (HAM-A > 7) and non-anxious (HAM-A ≤ 7) groups, and in the depressed (HAM-D > 7) and non-depressed (HAM-D ≤ 7) groups, statistical analyses were performed using *t*-tests or Mann-Whitney *U* tests. Correlations between EPVS characteristics and clinical scales (i.e., HAM-A and HAM-D) were analyzed using Pearson’s method if both continuous variables conformed to a normal distribution, otherwise, Spearman’s method was used. *P* values were adjusted by the false discovery rate (FDR) method. A two-tailed *adjusted_p* < 0.05 was considered statistically significant. To evaluate the classification performance of machine learning models, six quantitative metrics (i.e., AUC, accuracy, sensitivity, specificity, precision, and F1-score) were calculated. All statistical analyses were implemented using SPSS (version 26.0, https://www.ibm.com/spss) and R (version 4.2.2, https://www.R-project.org). All figures were plotted using Origin 2021 (https://www.originlab.com/), GraphPad Prism 9 (https://www.graphpad.com/), R (version 4.2.2), and Adobe Illustrator CC 2019 (https://www.adobe.com/products/illustrator.html).

## 3 Results

### 3.1 Participants characteristics

**Figure 2:**
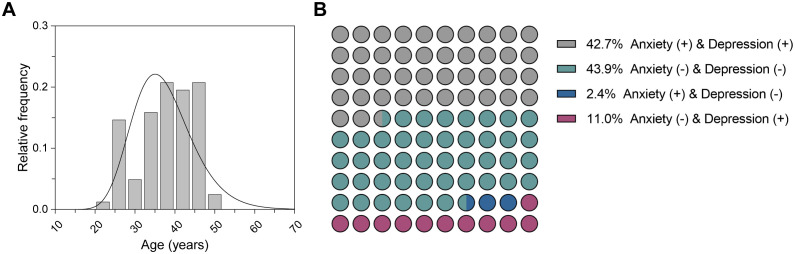
Characteristics of the participants. **(A)** Age distribution of all participants. **(B)** Distribution of anxiety and depression status for all participants.

We recruited 82 participants who underwent MRI examinations from the Affiliated hospital of Chengdu University of Traditional Chinese Medicine between October 2021 and May 2022. The demographics and clinical scales of each participant were collected and presented in **Table 1**. The median age of all participants was 38.0 years (**Figure 2**). Specifically, the median age of the non-anxious group (HAM-A ≤ 7) was 36.0 years, whereas the median age of the anxious group (HAM-A > 7) was 40.0 years, which was a significant difference between the two ages. There were no significant differences in sex distribution between the non-anxious and anxious groups, and between the non-depressed (HAM-D ≤ 7) and depressed (HAM-D > 7) groups. In addition, we observed a strong association between HAM-A status and HAM-D status. As shown in **Figure 2B**, up to 86.6% of the participants shared the same anxiety and depression status, revealing the intrinsic correlation between the two moods.

**Table 1:** Demographics of participants.

Variables	Overall (n = 82)	Non-anxiety (n = 45)	Anxiety (n = 37)	*P_(non-anxiety vs. anxiety)_*	Non-depression (n = 38)	Depression (n = 44)	*P_(non-depression vs. depression)_*
Age (years)	38.0 (33.0, 43.0)	36.0 (30.0, 42.0)	40.0 (35.0, 44.5)	0.045	36.0 (28.5, 43.0)	39.5 (34.0, 43.7)	0.096
Sex (M, %)	24 (29.3%)	17 (37.8%)	7 (18.9%)	0.062	11 (28.9%)	13 (29.6%)	0.953
HAM-A	6.0 (3.8, 14.2)	4.0 (2.0, 5.0)	15.0 (12.0, 18.5)	< 0.001	3.0 (1.0, 5.0)	13.0 (9.2, 18.0)	< 0.001
HAM-D	9.0 (4.0, 16.0)	5.0 (3.0, 8.0)	17.0 (12.5, 22.5)	< 0.001	4.0 (2.0, 6.0)	15.5 (11.0, 20.7)	< 0.001

To compare the distribution of characteristics between the non-anxiety and anxiety groups, Mann-Whitey *U* tests were used for continuous variables and the chi-square test was used for the categorical variable (i.e., sex). Similarly, comparison analyses were performed between the non-depression and depression groups. A two-tailed *P-value* < 0.05 was considered a significant difference.

### 3.2 Correlation analyses of EPVS characteristics and moods

**Figure 3:**
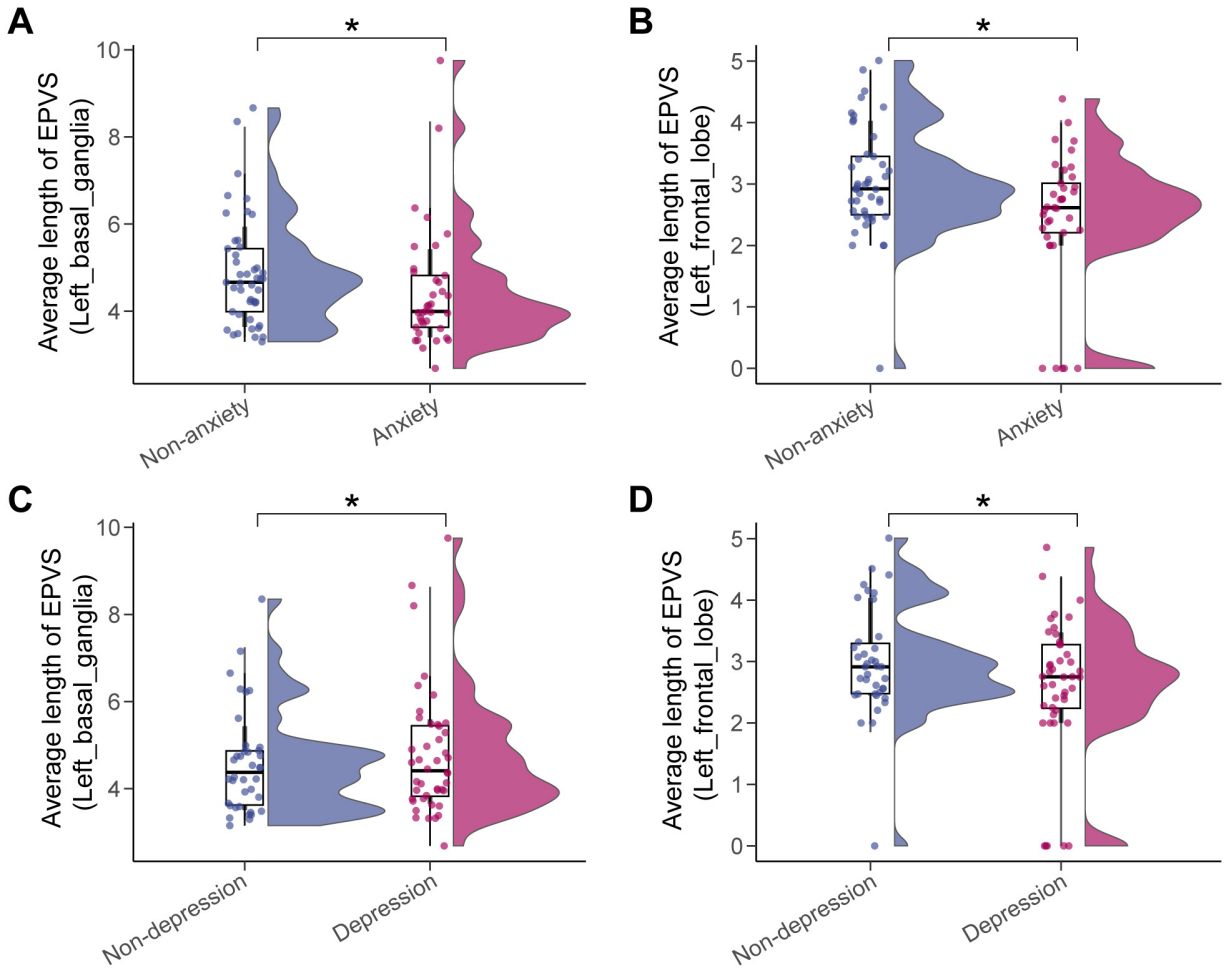
Significant differences in EPVS characteristics across mood status. The anxiety status affected the average length of EPVS lesions in the left basal ganglia **(A)** and the left frontal lobe **(B)**, as did depression status **(C, D)**. Asterisk represented a two-tailed adjusted *p-value*, with * indicating *adjusted_p* < 0.05.

A total of 70 EPVS quantitative features (i.e., total number, total volume, 4 features/subregion * 17 subregions) were extracted from each participant after brain parcellation on T1WI, EPVS definition on T2WI, and space alignment of the two modalities as detailed in Materials and Methods. The EPVS characteristics were compared between the non-anxious and anxious groups, and between the non-depressed and depressed groups, and the significant differences were summarized in **Supplementary Tables S2**, **S3**, and visualized in **Figure 3**. The average length of EPVS lesions in the left basal ganglia region and the left frontal lobe were smaller in patients in the anxiety group compared with the non-anxiety group (**Figures 3A, B**). There was also a significant difference between the depressed and non-depressed groups in terms of EPVS features in these two brain subregions, with smaller average length of EPVS lesions in the left frontal lobe and larger average length of EPVS lesions in the left basal ganglia region in the depressed group compared to the non-depressed group (**Figures 3C, D**). This suggests that there are similarities in EPVS feature performance between the two moods, but there are also some differences.

**Figure 4:**
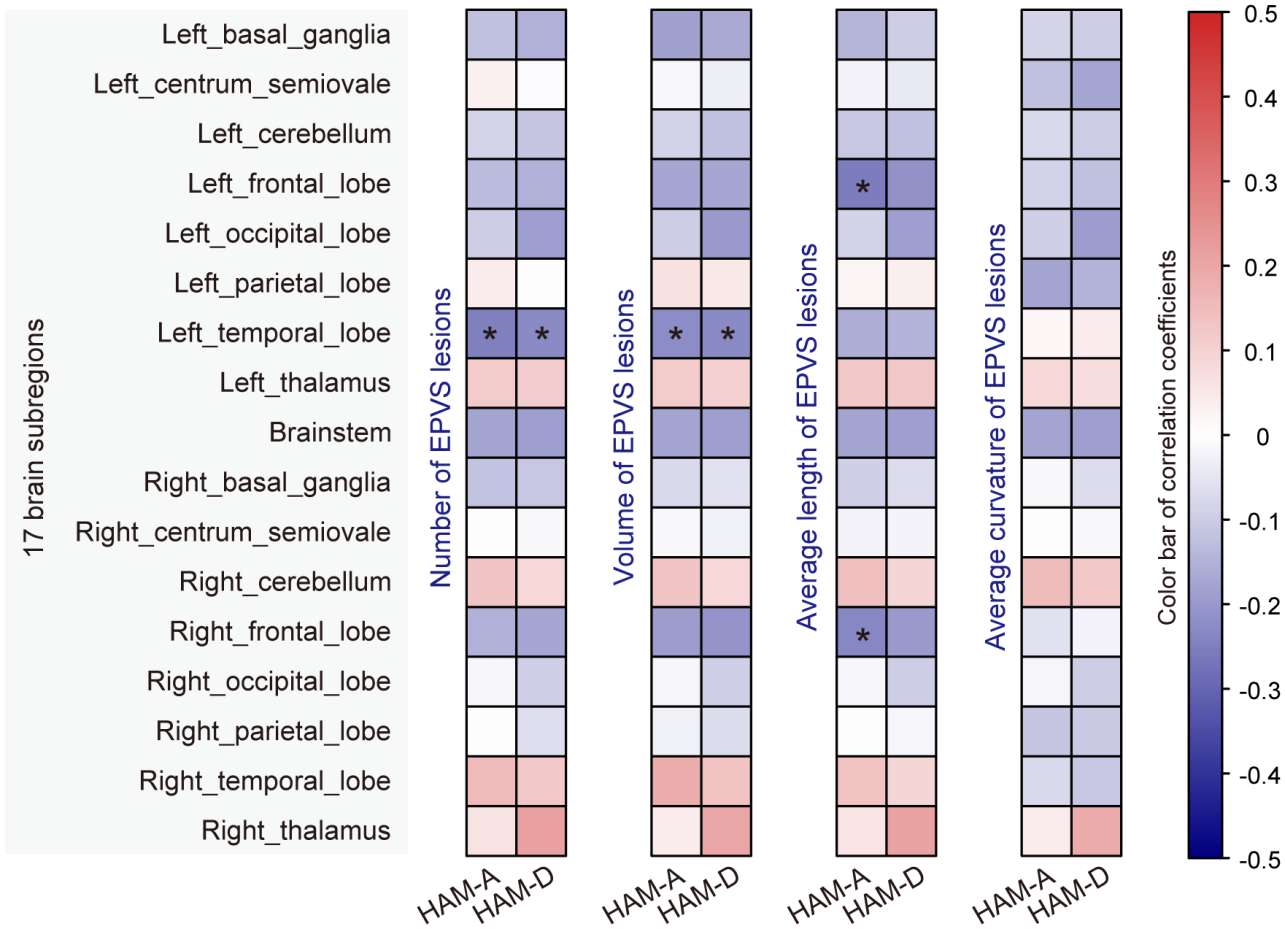
Correlation analysis of EPVS characteristics with mood scores. Correlation analyses were performed using Pearson’s or Spearman’s method, and significant correlations were marked with an asterisk (*adjusted_p* < 0.05).

In addition, we performed a correlation analysis between the EPVS characteristics and raw clinical scale scores (i.e., HAM-A score ranging from 0 to 56, and HAM-D score ranging from 0 to 52). As shown in **Figure 4**; **Supplementary Table S4**, EPVS characteristics in the left temporal, left frontal, and right frontal lobes were significantly correlated with mood scores. Specifically, the number and volume of EPVS lesions in the left temporal lobe were negatively correlated with both HAM-A and HAM-D scores. Moreover, the average length of EPVS lesions in the left frontal lobe and right frontal lobe were also negatively correlated with the HAM-A score.

### 3.3 Radiomics analysis predicting mood status

Given the clear correlation between EPVS features and mood scores/categories, we further explored the ability of EPVS features to predict mood status. A total of 70 EPVS features were combined with easily accessible participant demographics (i.e., sex, age) as inputs for selecting the most valuable features to build the machine learning model.

**Figure 5:**
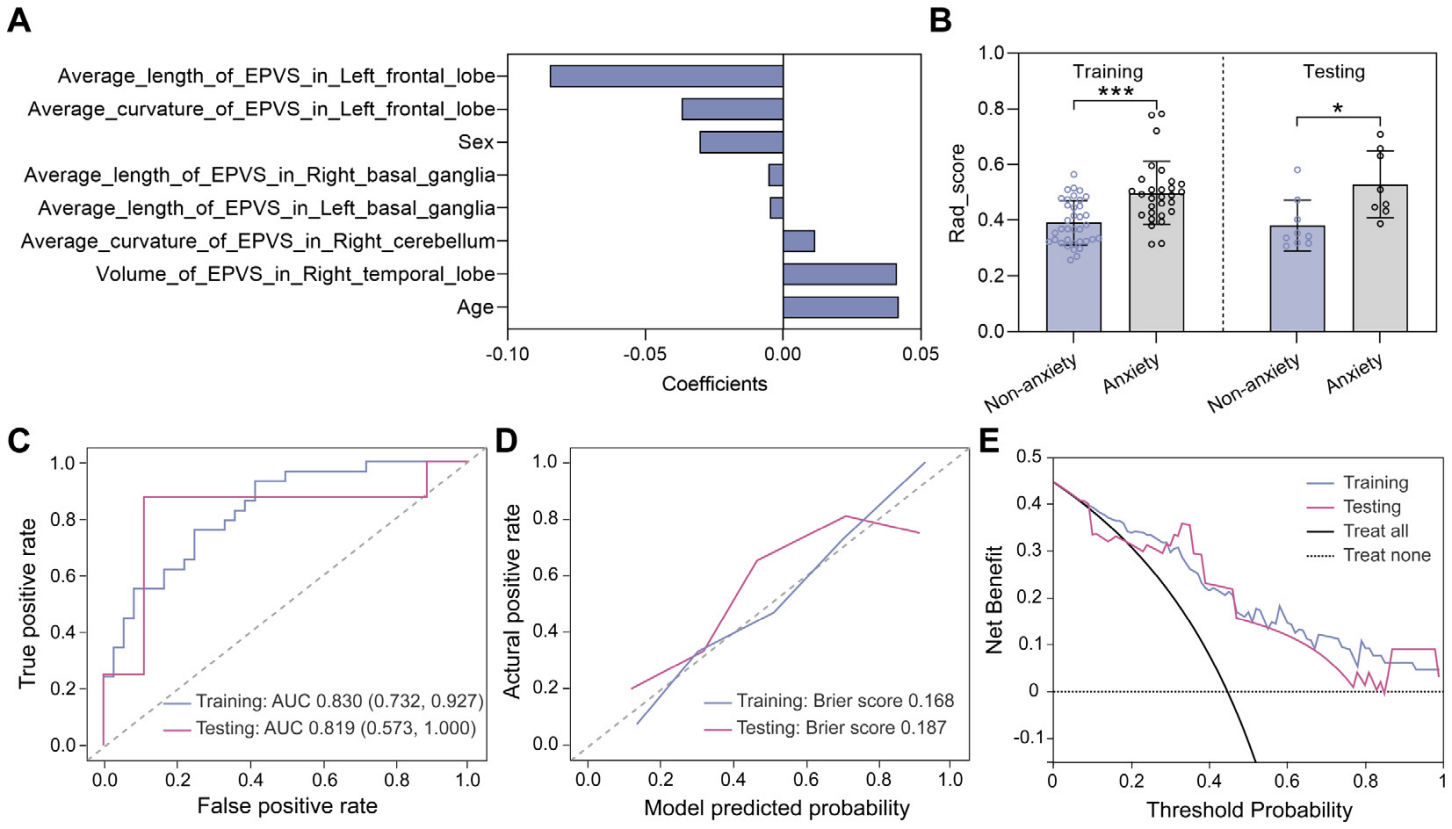
Model construction and evaluation in classifying anxiety status. **(A)** Eight features selected by the LASSO method. **(B)** Rad_score distributions of non-anxious and anxious groups in the training and testing datasets. Statistical analyses were performed using Mann-Whitney U tests. Asterisks represented two-tailed p values, with * indicating p<0.05, ** indicating p < 0.01, and *** indicating p<0.001. **(C)** ROC curves evaluating the trade-off between sensitivity and specificity of the LR model, with a higher AUC indicating a better discrimination ability of the model across different threshold settings. **(D)** Calibration curves evaluating the consistency of predicted probability and the actual HAM-A status. **(E)** Decision curves showing the clinical net benefit.

As mentioned above, the anxiety status could be classified into two categories based on HAM-A scores, with HAM-A ≤ 7 being the non-anxiety group and HAM-A > 7 being the anxiety group. For classifying anxiety status, eight features were selected using the LASSO method (**Figure 5A**), and a unique radiomics score (Rad_score) was calculated for each participant based on the feature values and the corresponding LASSO coefficients. As shown in **Figure 5B**, the Rad_score was higher for the anxious group compared to the non-anxious group in both the training and testing datasets, indicating a significant difference in the distribution of radiomics features between the two groups. Subsequently, a classification model was constructed using the logistic regression (LR) algorithm, with hyperparameters summarized in the **Supplementary Table S5**. Receiver operating characteristic (ROC) curves were plotted in **Figure 5C**. Specifically, the area under the ROC curve (AUC) values of the LR model were 0.830 with a 95% confidence interval (CI) of 0.732-0.927 and 0.819 (95% CI 0.573-1.000) in the training and testing datasets, respectively. The classification performance of the LR model was also evaluated by the other five quantitative metrics, as detailed in **Table 2**, calculated from the confusion matrix (**Supplementary Figure S2A**). It was easy to find that all metrics in the testing dataset were higher than 0.80, confirming that the LR model provided good classification performance. Additionally, the calibration curves demonstrated that the LR model fitted well with the actual anxiety status in the training dataset, and its performance was slightly degraded in the testing dataset (**Figure 5D**). Meanwhile, the LR model achieved a net clinical benefit in the threshold range of 0.3 to 0.7, implying that the model may be able to provide more meaningful clinical predictions by limiting the low false-positive rate while maintaining high sensitivity (**Figure 5E**).

**Table 2:** Performance of two machine learning models in predicting mood status.

	AUC (95% CI)	Accuracy	Sensitivity	Specificity	Precision	F1-score
LR for HAM-A classification						
• Training	0.830 (0.732, 0.927)	0.800	0.828	0.778	0.750	0.787
• Testing	0.819 (0.573, 1.000)	0.882	0.875	0.889	0.875	0.875
KNN for HAM-D classification						
Training	1.000 (1.000, 1.000)	1.000	1.000	1.000	1.000	1.000
Testing	0.931 (0.814, 1.000)	0.824	0.889	0.750	0.800	0.842

**Figure 6:**
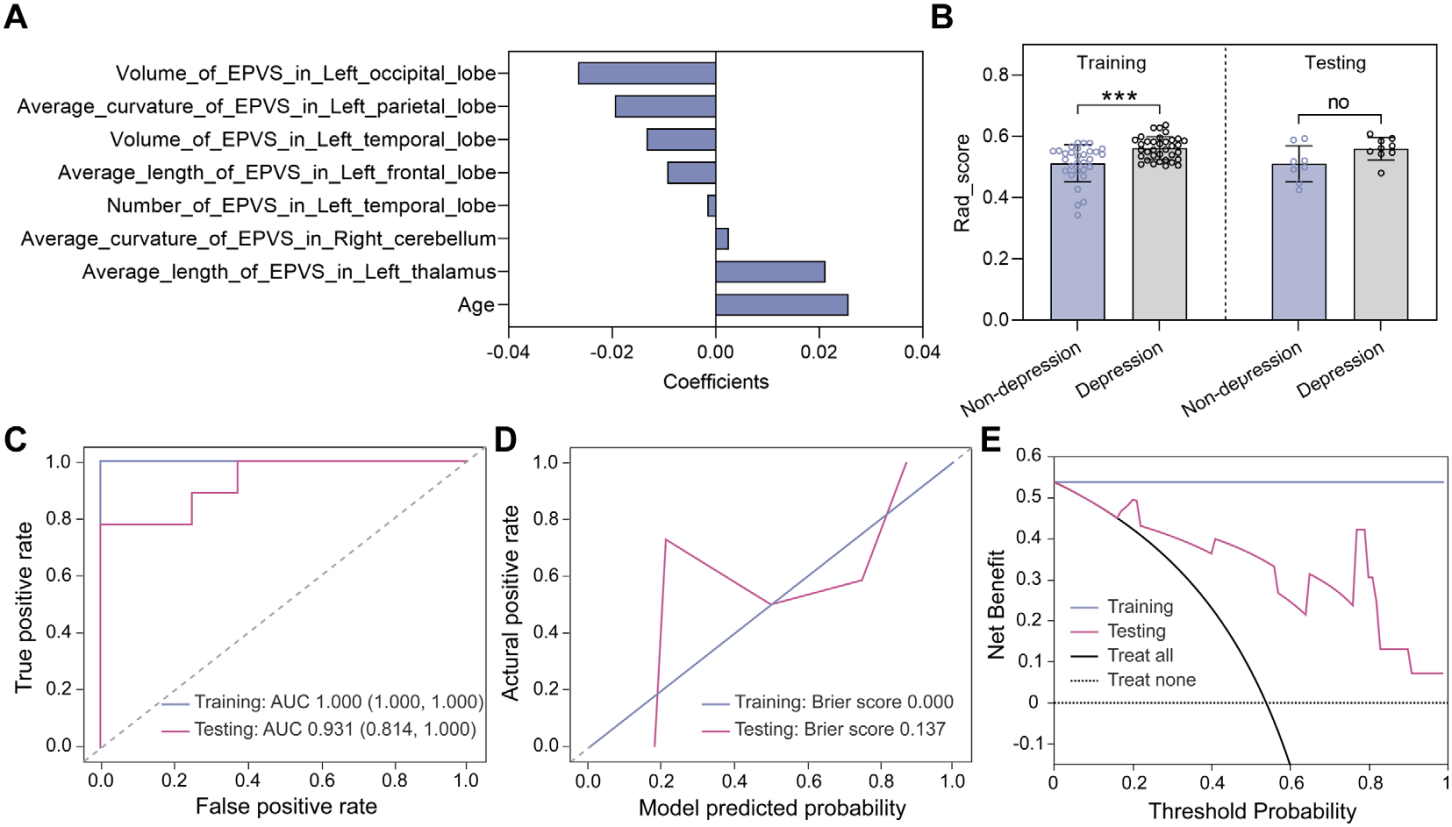
Model construction and evaluation in classifying depression status. **(A)** Eight features selected by the LASSO method. **(B)** Rad_score distributions of non-depressed and depressed groups in the training and testing datasets. Statistical analyses were performed using Mann-Whitney U tests. Asterisks represented two-tailed p values, with *** indicating p<0.001. **(C)** ROC curves, **(D)** calibration curves, and **(E)** decision curves were used to evaluate the model performance.

Similar feature selection and modeling procedures were performed to classify the non-depressed group (HAM-D ≤ 7) and the depressed group (HAM-D > 7). Eight features were selected using the LASSO method (**Figure 6A**) and the corresponding Rad_score was computed for each participant (**Figure 6B**). In the training dataset, the Rad_score of the depressed group was higher than that of the non-depressed group, whereas in the testing dataset, no significant difference was found, which may be due to the limited sample. Subsequently, the K nearest neighbors (KNN) algorithm was used to construct the classification model. As shown in **Figure 6C**; **Table 2**, the AUC values of the KNN model were 1.000 (95% CI 1.000-1.000) and 0.931 (95% CI 0.814-1.000) in the training and testing datasets, respectively. Meanwhile, the KNN model achieved an accuracy of 100.0% and 82.4% in the training and testing datasets, respectively, demonstrating its excellent classification performance. Moreover, the calibration curves demonstrated good alignment between the KNN-predicted probabilities and actual depression status (**Figure 6D**). The decision curves plotted in **Figure 6E** showed that the KNN model achieved high clinical net benefit across a broad range of thresholds (0.2-1.0), demonstrating its potential to effectively contribute to improved patient care and decision support.

## 4 Discussion

To our knowledge, this paper is the first study that presents a novel approach to classify anxiety and depression symptom severity in young adults with LTMPU by integrating MRI-based quantification of EPVS and machine-learning algorithms. Our model demonstrated significant accuracy in classifying anxiety and depression severity, offering a promising avenue for non-invasive, objective assessment. Furthermore, our study provides evidence indicating a relationship between EPVS and the severity of anxiety symptoms, an aspect that, as far as we know, has received limited attention in previous research.

EPVS and cerebrospinal fluid are integral components of the glymphatic system, which is responsible for maintaining brain homeostasis and clearing neural waste throughout the lifespan (24). The clearance of cellular byproducts associated with mental health and neurodegenerative processes, such as amyloid-β and tau, is partly dependent on the integrity of the glymphatic function (25). EPVS visibility serves as a proxy measure of glymphatic dysfunction and potential occlusion of drainage pathways (26). It is plausible to hypothesize that EPVS may be associated with mental illness, including anxiety and depression, although the evidence base is limited (27). Previous studies have demonstrated that EPVS are risk factors for incident depression and are associated with depressive symptoms in the general population (28). MRI is the gold standard for *in vivo* assessment of EPVS (26). Quantitative assessment of EPVS severity using grading scales offers high reproducibility and convenience but lacks precision for nuanced analysis and longitudinal studies (29, 30). Recent advancements in automated segmentation algorithms have enabled volumetric and morphological analysis of EPVS, mitigating inconsistencies observed with different grading methods (30). In our previous study, we have applied VB-Net to segment EPVS, and the recall and precision reached 0.953 and 0.923, respectively, highlighting the model’s reliability in accurately identifying and quantifying EPVS (21). Notably, the VB-Net incorporates the advantages of an efficient encoder-decoder framework for feature embedding, residual connections for information flow, and bottleneck layers for model compression. The detailed network architecture can be found in our previously published paper (31).

Our findings indicate a potential glymphatic biomarker of anxiety symptom, evidenced by the reduced average length of EPVS lesions in the left basal ganglia region and the left frontal lobe. This segment of our findings are in conceptual accordance with existing literature, highlighting individual variability in relative left frontal electroencephalographic (EEG) activity that may correlate distinctively with specific symptom clusters of depression (i.e., anhedonia), and anxiety (i.e., anxious apprehension versus anxious arousal) (32). Neuroimaging studies have implicated structural modifications within regions fundamental to threat response in anxiety disorders, particularly within the medial temporal, prefrontal cortex, and cingulate regions (6). However, due to the scarcity of research in this domain, it remains uncertain whether the structural changes of basal ganglia detected by neuroimaging method is associated with anxiety symptoms. Paradiso et al. posited that the basal ganglia play a pivotal role in emotion perception (33). We hypothesize that the observed reduction of EPVS lesion length in the left basal ganglia may be related to emotional disturbances, specifically anxiety symptom, encountered by young adults with LTMPU. Further investigation is warranted to substantiate the correlation between these structural alternations and the emotional manifestations found in this demographic.

We further found that young adults with depressive symptoms have smaller average length of EPVS lesions in the left frontal lobe and larger average length of EPVS lesions in the left basal ganglia. Functional neuroimaging studies have consistently illuminated the neural substrates of depression, revealing impairments in emotion regulation, the tendency toward rumination, disruptions in reward pathways associated with anhedonia, and alterations in self-awareness among individuals (34). Our finding of the left frontal lobe is consistent with recent advancements in the field, which have highlighted the hypoactive state of the insula and dorsal lateral prefrontal cortex in those with depression (35). A meta-analysis in unipolar depression have found that volumetric reductions within the basal ganglia, including both caudate nucleus and putamen, are characteristic of this condition, with the putamen exhibiting a more pronounced effect size (36). Our study introduces a novel finding that diverges from this narrative, indicating an enhancement in glymphatic function specifically within the left basal ganglia. The juxtaposition of these findings accentuates the intricate interplay between structural integrity and functional dynamics within the basal ganglia and their implications for depressive symptomatology. It underscores an urgent need for further inquiry to demystify the precise mechanisms at work within these structures that contribute to the experience of depression.

A prior study involving 4,333 participants with an 8-month follow-up period identified the predictive impact of LTMPU on the incidence of new cases of depression and anxiety. It also revealed bidirectional longitudinal relationships between the duration of mobile phone use and the severity of these conditions (4). An accurate assessment of anxiety and depression severity is crucial for effective treatment planning (6, 7). Building upon the growing use of machine learning techniques for differentiating anxiety and depression severity (37), this study represents a first attempt to apply this approach specifically to young adults with LTMPU, which may hold significant potential for clinical applications. By integrating MRI-quantified EPVS volumes with machine learning algorithms, our model is capable of extracting valuable information about glymphatic dysfunction that is challenging to detect through traditional methods. This approach may generalize to assess the severity of anxiety disorders and depression, thereby serving as an auxiliary tool for psychiatrists. It can assist in accurately gauging the severity of these conditions in patients, aiding in developing more personalized treatment plans and facilitating the monitoring of treatment progress.

This study is subject to several limitations. Firstly, the relatively small sample size potentially impacts the precision of the model, underscoring the necessity for larger datasets to enhance generalizability. Moreover, the single-center nature of the study necessitates further validation across diverse populations.

## 5 Conclusions

This study demonstrates the feasibility and potential of utilizing MRI-quantified EPVS volumes and machine-learning algorithms to classify anxiety and depression symptom severity in young adults with LTMPU. The proposed model offers a valuable tool for clinicians, providing a non-invasive, objective, and quantitative approach to enhance diagnostic efficiency and guide personalized treatment strategies. Future research should focus on validating the model across various populations and exploring the inclusion of EPVS location analysis to enhance its clinical utility.

## Data Availability

The original contributions presented in the study are included in the article/**Supplementary Material**. Further inquiries can be directed to the corresponding authors.
