# Supplementary material for: MRI quantified enlarged perivascular space volumes as imaging biomarkers correlating with severity of anxiety depression in young adults with long-time mobile phone use
Source: Front Psychiatry. 2025 Feb 20;16:1532256. doi: 10.3389/fpsyt.2025.1532256 (PMC11882520; doi:10.3389/fpsyt.2025.1532256)
Supplement: Supplementary file 1 [file DataSheet1.pdf]

Supplementary Information

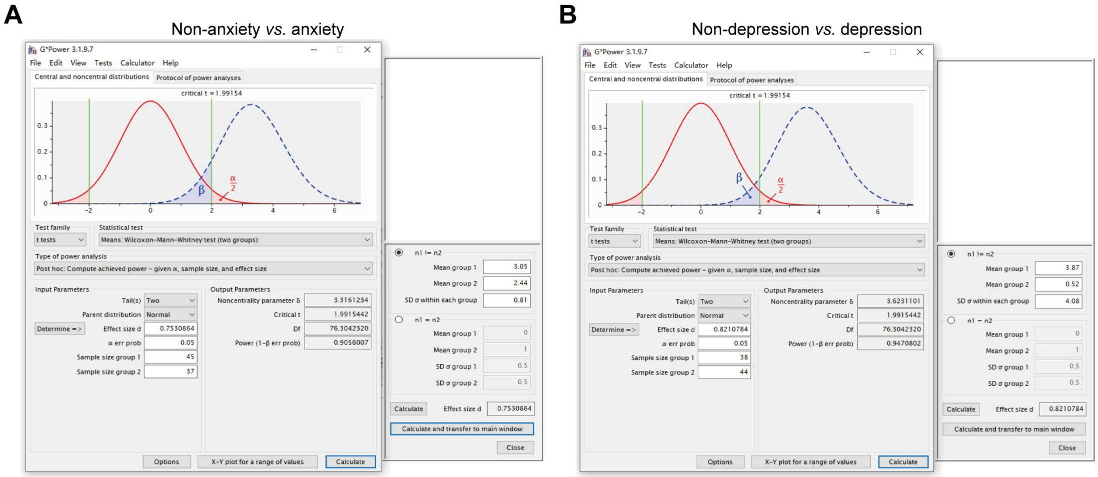

Supplementary Figure 1. Statistical power calculated by G*Power software. (A) The Average_length_of_EPVS_in_left_frontal_lobe feature was used to quantify the difference between the non-anxiety and anxiety. The power reached 0.906 for the given parameters (α , sample sizes, means, and σ). **(B)** The Volume_of_EPVS_in_left_occipital_lobe feature was used to quantify the difference between the non-depression and depression. The power reached 0.947 for the given parameters (α, sample sizes, means, and σ).

Supplementary Table 1. A total of 17 brain subregions were obtained by automatic brain parcellation and merging neighboring anatomical structures. Other anatomical structures including Cerebral_WM, Corpus_Callosum, and Cingulate_Gyrus were divided into frontal, parietal, occipital, and temporal lobes according to the principle of proximity.

Number	Brain subregions	Anatomical structures
1	Frontal_Lobe_L	Precentral_L, Frontal_Sup_L, Frontal_Mid_Rostral_L, Frontal_Mid_Caudal_L, Frontalpole_L, Orbitofrontal_Lat_L, Orbitofrontal_Med_L, Parsopercularis_L, Parsorbitalis_L, Parstriangularis_L
2	Frontal_Lobe_R	Precentral_R, Frontal_Sup_R, Frontal_Mid_Rostral_R, Frontal_Mid_Caudal_R, Frontalpole_R, Orbitofrontal_Lat_R, Orbitofrontal_Med_R, Parsopercularis_R, Parsorbitalis_R, Parstriangularis_R
3	Parietal_Lobe_L	Postcentral_L, Paracentral_L, Parietal_Sup_L, Parietal_Inf_L, Precuneus_L, Supramarginal_L
4	Parietal_Lobe_R	Postcentral_R, Paracentral_R, Parietal_Sup_R, Parietal_Inf_R, Precuneus_R, Supramarginal_R
5	Occipital_Lobe_L	Cuneus_L, Lingual_L, Pericalcarine_L, Occipital_Lat_L
6	Occipital_Lobe_R	Cuneus_R, Lingual_R, Pericalcarine_R, Occipital_Lat_R
7	Temporal_Lobe_L	Hippocampus_L, Parahippocampal_L, Entorhinal_L, Fusiform_L, Temporal_Sup_L, Temporal_Mid_L, Temporal_Inf_L, Temporalpole_L, Temporal_Sup_Banks_L,

		Transversetemporal_L
8	Temporal_Lobe_R	Hippocampus_R, Parahippocampal_R, Entorhinal_R, Fusiform_R, Temporal_Sup_R, Temporal_Mid_R, Temporal_Inf_R, Temporalpole_R, Temporal_Sup_Banks_R, Transversetemporal_R
9	Cerebellum_L	Cerebellum_Cortex_L, Cerebellum_WM_L
10	Cerebellum_R	Cerebellum_Cortex_R, Cerebellum_WM_R
11	Thalamus_L	Thalamus_L, VentralDC_L
12	Thalamus_R	Thalamus_R, VentralDC_R
13	Basal_Ganglia_L	Amygdala_L, Caudate_L, Putamen_L, Pallidum_L, Accumbens_Area_L, Insula_L
14	Basal_Ganglia_R	Amygdala_R, Caudate_R, Putamen_R, Pallidum_R, Accumbens_Area_R, Insula_R
15	Centrum_semiovale_L	Centrum_semiovale_L
16	Centrum_semiovale_R	Centrum_semiovale_R
17	Brainstem	Pons, Midbrain, Medulla, SCP

Supplementary Table 2. Significant differences of EPVS characteristics between the non-anxious and anxious groups. Continuous variables were compared using *t* tests or Mann-Whitney *U* tests, with *p* values adjusted by false discovery rate (FDR) method, retaining only those variables with an *adjusted_p* < 0.05.

Variables	Non-anxiety (n = 45)	Anxiety (n = 37)	*Adjusted_p*
Average length of EPVS in Left_basal_ganglia	4.66 (3.99, 5.44)	3.99 (3.63, 4.82)	0.036
Average length of EPVS in Left_frontal_lobe	2.92 (2.50, 3.45)	2.61 (2.21, 3.01)	0.019

Supplementary Table 3. Significant differences of EPVS characteristics between the non-depressed and depressed groups. Continuous variables were compared using *t* tests or Mann-Whitney *U* tests, with *p* values adjusted by FDR method, retaining only those variables with an *adjusted_p* < 0.05.

Variables	Non-depression (n = 38)	Depression (n = 44)	*Adjusted_p*
Average length of EPVS in Left_basal_ganglia	4.38 (3.62, 4.87)	4.41 (3.83, 5.44)	0.036
Average length of EPVS in Left_frontal_lobe	2.91 (2.48, 3.29)	2.75 (2.24, 3.28)	0.020

Supplementary Table 4. Significant correlations between the EPVS characteristics and clinical scale scores. Correlation analyses were performed using Pearson's or Spearman's methods, and p values were adjusted by the FDR method, retaining only pairs with *adjusted_p* < 0.05.

EPVS features	Scales	Coefficient	*Adjusted_p*
Volume in Left_temporal_lobe	HAM-A	-0.22	0.043
Number in Left_temporal_lobe	HAM-A	-0.25	0.026
Average length in Left_frontal_lobe	HAM-A	-0.25	0.022
Average length in Right_frontal_lobe	HAM-A	-0.23	0.037
Volume in Left_temporal_lobe	HAM-D	-0.23	0.038
Number in Left_temporal_lobe	HAM-D	-0.23	0.039

Supplementary Table 5. Hyperparameters used by the two models.

Classification task	Machine learning algorithm	Hyperparameters
Anxious status (HAM-A > 7 *vs.* HAM-A ≤ 7)	LR	• Regularization parameter C: 1.0<br>• Class weight: None<br>• Penalty parameter: l2<br>• Tolerance: 0.0001<br>• Classification threshold: 0.45
Depression status (HAM-D > 7 *vs.* HAM-D ≤ 7)	KNN	• Algorithm: ball trees<br>• Leaf size: 30<br>• Number of neighbors (k): 5<br>• Parallelization parameters: 2<br>• Distance weights

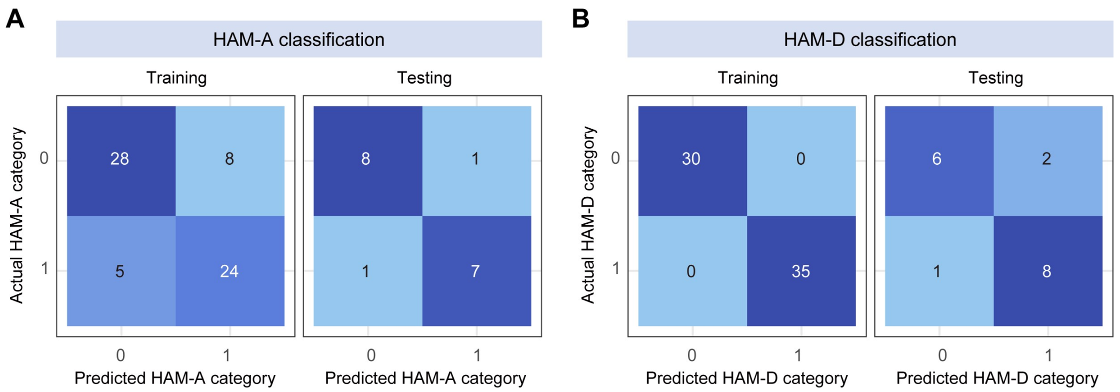

Supplementary Figure 2. Confusion matrices of models for classifying (A) HAM-A status and (B) HAM-D status in the training and testing datasets.
